# Fatigue in living kidney donors compared to a German general population sample: an exploratory study

**DOI:** 10.3389/fpsyt.2024.1510738

**Published:** 2025-01-30

**Authors:** Dilek Akkus, Adrian Westenberger, Gunilla Einecke, Wilfried Gwinner, Uwe Tegtbur, Mariel Nöhre, Martina de Zwaan

**Affiliations:** ^1^ Department of Psychosomatic Medicine and Psychotherapy, Hannover Medical School, Hannover, Germany; ^2^ Department of Nephrology and Rheumatology, University Medical Center Göttingen, Göttingen, Germany; ^3^ Department of Nephrology and Hypertension, Hannover Medical School, Hannover, Germany; ^4^ Department of Rehabilitation and Sports Medicine, Hannover Medical School, Hannover, Germany

**Keywords:** living kidney donation, fatigue, MFI-20, sex, age, education, general population sample

## Abstract

**Background:**

Clinical studies have not conclusively clarified whether fatigue scores in living kidney donors after donation are fundamentally different from general population samples. Moreover, the association between sociodemographic and donor specific factors and fatigue in donors is not well understood.

**Patients and methods:**

Fatigue scores of 358 living kidney donors on average 7.67 years post-donation were compared with 1896 subjects from the German general population in five strata of age and sex. Fatigue was measured with the Multidimensional Fatigue Inventory (MFI-20). Relationships between the five MFI-20 subscales and the sociodemographic variables sex, age, education, and in the donor sample also years since donation were calculated. Additionally, the association between donor specific variables and fatigue levels were analyzed.

**Results:**

Overall, donors had lower fatigue scores than the population sample. Particularly the age group 65-74 and above reported significantly lower fatigue scores. A significant exception was found in women aged 45-54 years, where donors showed significant higher general fatigue scores than the corresponding subgroup of the general population sample. Multiple regression analyses in the general population sample revealed associations between female sex and higher age with higher values in most MFI-20 subscales, whereas subjects with higher education showed mostly lower fatigue scores. In the donor group, these associations were of little importance. Also, years since donation, partnership, and recipient group were not strongly related to fatigue. However, higher fatigue in donors was associated with more donation regret, a more negative relationship with the recipient, a more negatively perceived recipient health, less perceived family support, and more financial burden.

**Conclusion:**

Fatigue is less prevalent particularly in older donors and predictors of fatigue presented in the general population sample seem to have little importance in the donors. However, middle-aged female donors might be more prone to develop fatigue. This group may require more intense exploration before and after donation to detect and treat the underlying factors timely.

## 1 Introduction

Currently, in Germany, over 8,000 people are waiting for a donor organ, with the waiting time for a kidney being on average eight to ten years (1). In contrast to other European countries such as Spain, the number of organ donors in Germany is comparatively low, resulting in limited organ availability. Kidney transplantation is one of the few transplantation types that can be performed using a living donor and is becoming increasingly important in the German healthcare system. In 2022, 1,966 kidney transplants were performed in Germany, of which 535 (27.21%) were from living donors (1). A close emotional bond between donor and recipient is required by German law, and non-directed altruistic donation is prohibited (2). These legal restrictions limit the pool of potential living donors and may lead to different donor collectives compared to studies conducted in other countries. Globally, kidney transplantation is the optimal treatment for most patients with end-stage renal disease (ESRD), which is a growing health problem (3, 4). More than 20,000 living kidney transplants are performed each year (5). The proportion of living kidney donations differ significantly between different countries (3).

Although potential living kidney donors (LKD) in Germany undergo extensive physical and psychosocial evaluations, living donation is not a risk-free intervention. LKD have only a slightly increased risk of developing severe chronic kidney disease (CKD) and end-stage renal disease (ESRD).

Fatigue following surgery, including donor nephrectomy, is common and clinically expected (4, 6–11). While most LKD return to baseline fatigue scores several months after surgery (6, 12), some show persistently elevated fatigue scores compared to their pre-donation scores (e.g., 1 month, 6 months,12 months or up to 24 months post-donation) (6–8, 10, 13, 14), even when the overall post-donation fatigue scores are still comparable to control samples. Rodrigue et al. (6) reported persistent clinical fatigue lasting 24 months post-donation in 12% of their sample of 189 LKD. A recently published multicenter study in Germany reported significantly higher postoperative mental fatigue scores in LKD compared to the general population twelve months after donation (10) while the mean level of mental fatigue before donation was lower than in the German general population. This is in line with several studies showing that LKD normally represent a very healthy population (15). Unfortunately, data on fatigue as a putative complication of LKD are still rare. Furthermore, there is a lack of age and sex-based comparisons of LKD with non-donors which limits conclusive statements concerning the comparison with population-based studies (13, 16). Moreover, inconclusive results have been reported regarding the associations of socioeconomic variables such as age and sex with fatigue scores in LKD. For example, negative (7) or no associations (4, 9) between age and fatigue scores, and positive (4, 16) or no associations (6) between female sex and fatigue scores have been found. More research on factors affecting fatigue in LKD is considered necessary (8).

This study aims to provide an explorative overview of fatigue levels in LKD compared to a general population sample, highlighting differences across subgroups based on age and sex and thus may contribute to a better understanding of fatigue in this population. The fatigue scores of a large German sample of LKD were compared with those of a representative German population sample by age and sex, using the Multidimensional Fatigue Inventory (MFI-20). Additionally, the relationship between the MFI-20 sum score its five dimensions and the sociodemographic variables sex, age, education, and in the donor sample also years since donation was calculated. The association between donor specific variables with fatigue levels were examined including relationship quality with the recipient, recipient group, partnership status, financial situation, feeling of having been pushed, regret of donation, decision to donate, reaction of the family after donation, and perceived health status of the recipient were examined. Furthermore, based on previous studies (10) we also investigated potential thresholds for clinically relevant fatigue by comparing donor values with standardized thresholds from the general population sample.

## 2 Material and methods

### 2.1 Sampling

The LKD dataset is based on a sample size of 361, collected from the Hannover Medical School database between July 30th, 2016, and July 1st, 2017. The inclusion criterion was a time span of at least one year since the date of donation (1978-2016) (17, 18). Paper-pencil surveys were carried out to collect information on donor demographics, donor specific variables, as well as several standardized questionnaires, including the Multidimensional Fatigue Inventory (MFI-20) (19, 20). After contacting donors up to three times, 60% responded. The ethics committee of the Hannover Medical School approved the study with all patients giving written informed consent (no 3252-2016).

A sample of the German population with 2509 participants serves as the comparison sample in this study. The participants were recruited via a market and social research institute (USUMA GmbH, Berlin, Germany) in the time period between October 2nd and December 12th 2021. The study population is a random sample, stratified by sex, age, and regional distribution (16 federal states), of citizens aged 16 years or older, and officially registered in Germany. The dataset includes socio-demographic variables and other standardized questionnaires through face-to-face surveys by trained interviewers as well as self-reported paper-pencil questionnaires, such as the MFI-20. Informed consent was obtained from all respondents in accordance with the Declaration of Helsinki. The study was approved by the ethics committee of the University of Leipzig (298/21-ek). Further details on the samples can be found in previous papers (17, 18, 20). This study focuses on the results of the fatigue questionnaire MFI-20.

### 2.2 Measurements

#### 2.2.1 Multidimensional Fatigue Inventory (MFI-20)

The Multidimensional Fatigue Inventory (MFI-20) (19) is one of the most commonly used instruments in Europe to assess fatigue and has been used in several studies to explicitly measure fatigue after living kidney donation (11, 13, 14, 21, 22). The MFI-20 comprises 20 items covering the five subscales of general fatigue, physical fatigue, mental fatigue, reduced motivation, and reduced activity. These dimensions refer to subscales which have been formed by factor analyses (19). Fatigue can be expressed by a person’s general statement about their functioning, such as “I feel rested” (general fatigue) as well as by referring to physical sensations related to the experience of fatigue (physical fatigue). The term fatigue is also used to describe indications of cognitive symptoms such as difficulties in concentration (mental fatigue), a lack of motivation to engage in activities (reduced motivation) and impairments in a person’s activity (reduced activity) (19). Each subscale consists of four items with response options on a five-point Likert scale from one to five (ranging from completely affirmative to completely incorrect). The range of the scales are 4 to 20 for the subscales and 20 to 100 for the MFI sum score. Higher scores indicate more pronounced fatigue levels. In collaboration with the developers of the MFI-20, the original English version of this scale was translated into German by independent translators (23). Westenberger and colleagues (20) report that the MFI-20 reflects satisfactory psychometric properties, including reliability and convergent validity, based on the general German population sample.

#### 2.2.2 Donor-specific questions

The participants also responded to donor-specific questions, measuring factors such as economic situation (mainly negative/unchanged/mainly positive), regret of donation (not at all/some doubt), having felt pressured to donate (not at all/at least somewhat), decision to donate (easy/neutral/difficult), perceived recipient outcome (mainly negative/neutral/mainly positive), relationship (mainly negative/neutral/mainly positive) and change in relationship quality with the recipient after donation (mainly negative/neutral/mainly positive), recipient group (spouse/family/other), and recognition from family after donation (mainly negative/neutral/mainly positive).

### 2.3 Data processing

Data analyses and data processing were conducted using R (version 4.2.2). Reverse items were inverted prior to further analyses to ensure that higher scores consistently indicate higher levels of fatigue across all items. As recommended in the work of Schwartz et al. (22), sum scores of the five MFI-20 subscales and a total MFI-20 sum score were calculated. Due to the previously reported age and sex dependence of fatigue scores, subgroup comparisons were conducted to examine differences in fatigue scores between subjects of similar age and sex (4, 22). Following Westenberger et al. (20), seven age groups (≤ 24, 25 - 34, 35 - 44, 45 - 54, 55 - 64, 65 - 74, and > 74 years) were created for both samples, divided by self-reported sex (male and female). As there were no cases in the donor population under 24 years of age and only three cases aged 25-34 years, these two age groups were excluded from the analysis in both samples (**Supplementary Table S1**). The data of the general population sample includes three individuals of diverse gender, who were also excluded from all analyses. The final datasets consisted of 358 LKD and 1896 individuals from the general population. MFI-20 sum scores were calculated only when complete data were available within the subscales and the sum score. Subjects with missing items within one subscale, however, were not automatically excluded from further subscale comparisons, provided that complete scores were available for those subscales. The exact response frequencies are reported in **Appendix Supplementary Table S2**. Overall, the rate of missing values was low.

Based on the datasets used in this study we furthermore calculated how many LKD had fatigue scores exceeding one or two standard deviations of the mean fatigue scores in the general population sample for each MFI-20 subscale (**Supplementary Table S8**), which can be seen as a starting point for deriving cut-offs for clinically relevant fatigue scores in future studies. In the study by Suwelack et al. (10), it was assumed that clinically relevant fatigue symptoms were present if the fatigue score was more than one standard deviation above the score of the German general population sample. It is crucial to note that this definition may lead to overestimation of clinical significance. Therefore, we decided to perform the calculation in addition with two standard deviations to mitigate the risk of overestimating the clinical significance.

### 2.4 Statistical analysis

Differences in the individual subscales between donors and the general population sample were compared by age and sex (between group analyses). Since the MFI-20 scores were not normally distributed and the donor population and the general population sample were unequal in sample size, the Wilcoxon-Mann-Whitney U test was performed. Effect sizes were calculated using Cohen’s d with 95% confidence interval and were interpreted according to Cohen (24): d >.20 (weak effect), d >.50 (medium effect), d >.80 (large effect), as the group sizes differ strongly from each other.

In addition, two multiple linear regression analyses were conducted separately for the donor sample and the general population sample to disentangle the associations between sociodemographic variables and the MFI-20 scores (within-group analyses). The sociodemographic variables age, sex, education and, for the donor sample, years since donation, were used as independent variables. Age and years since donation were numerical variables, sex and education were binary categorical. For the variable education (donor sample: 5 levels, general population sample: 9 levels), the highest attained school education was dichotomized into A-level (yes/no). In addition to the main analyses, interaction effects between age and time since donation were calculated for the MFI-20 subscales to investigate potential interactions between the variables age and years since donation on fatigue scores.

The regression coefficient β was used to calculate the effect of these variables on the MFI-20 subscales and the MFI-20 sum score. The significance level for all analyses was consistently set at 5% (two-sided) without adjusting for multiple tests as this study is exploratory. The coefficients of determination R² were evaluated according to Cohen (24): weak variance explanation |R²| = .02, moderate variance explanation |R²| = .13, strong variance explanation |R²| = .26.

Additionally, univariate non-parametric analyses were conducted to examine the association between donor specific questions and fatigue levels in LKD.

## 3 Results

### 3.1 Characteristics of the LKD and the general population sample

Donors who did not return the survey did not differ significantly with regard to age [55.0 (SD 8.4) versus 55.9 (SD 8.0) years], sex (females 65.9% versus 64.1%), and organ recipient group when compared with donors who returned the survey. Duration since donation was longer in participants compared to non-participants [8.24 (SD 5.2) versus 7.1 (SD 5.2) years; *T* = −2.297 (df = 477), *p* = 0.022] (18).

N = 128 (35.75%) of the LKD were male and n = 230 (64.25%) were female (descriptive statistics **Table 1**). The mean age at donation was 58.57 years (SD 9.71), and the mean time since donation was 7.67 years (SD 5.72). **Supplementary Table S3** provides more detailed information on the variable years since donation and on the kidney recipient for the donor subgroups. The time since donation is generally longer for older than for younger donors. Years since donation between male and female donors within the age groups are comparable. Most kidney recipients were daughters/sons of the donors [n = 162 (44.88%)], followed by spouses [n = 135 (37.40%)] and siblings [n = 38 (10.53%)], (**Supplementary Table S3**). Of the LKD, 91 (25.42%) reported having a high school diploma (A-level), while n = 266 (74.30%) had other qualifications or no school diploma at all.

**Table 1:** Characteristics of the German general population sample, and the LKD sample for the between group analysis.

	Total sample	General Population sample	LKD sample
Age distribution (at the time of investigation)			
n	2254	1896	358
M	57.29	57.05	58.57
SD	12.77	13.25	9.71
Range (Min, Max)	35-95	35-95	35-87
IQR	48-67	46-67	52-66
Median	57	56	58
Sex			
Male	1069 (47.43%)	941 (49.63%)	128 (35.75%)
Female	1185 (52.57%)	955 (50.37%)	230 (64.25%)
Education level			
A-level	485 (21.52%)	394 (20.78%)	91 (25.42%)
No A-level	1768 (78.44%)	1502 (79.22%)	266 (74.3%)
NA	1 (0.04%)	NA	1 (0.28%)
Years since donation			
M			7.67
SD			5.72
Range (Min, Max)			0-38
IQR			3.25-11
Median			7

IQR, interquartile range; M, mean; SD, standard deviation.

The general population sample consists of 941 (49.63%) males and 955 (50.37%) females with an overall mean age of 57.05 years (SD 13.25). Similar to the donor group, at least n = 394 (20.78%) of the subjects have a high school diploma (A-level), while 1502 (79.22%) report having another or no school diploma.

### 3.2 Comparison between the LKD and the general population sample

**Figure 1:**
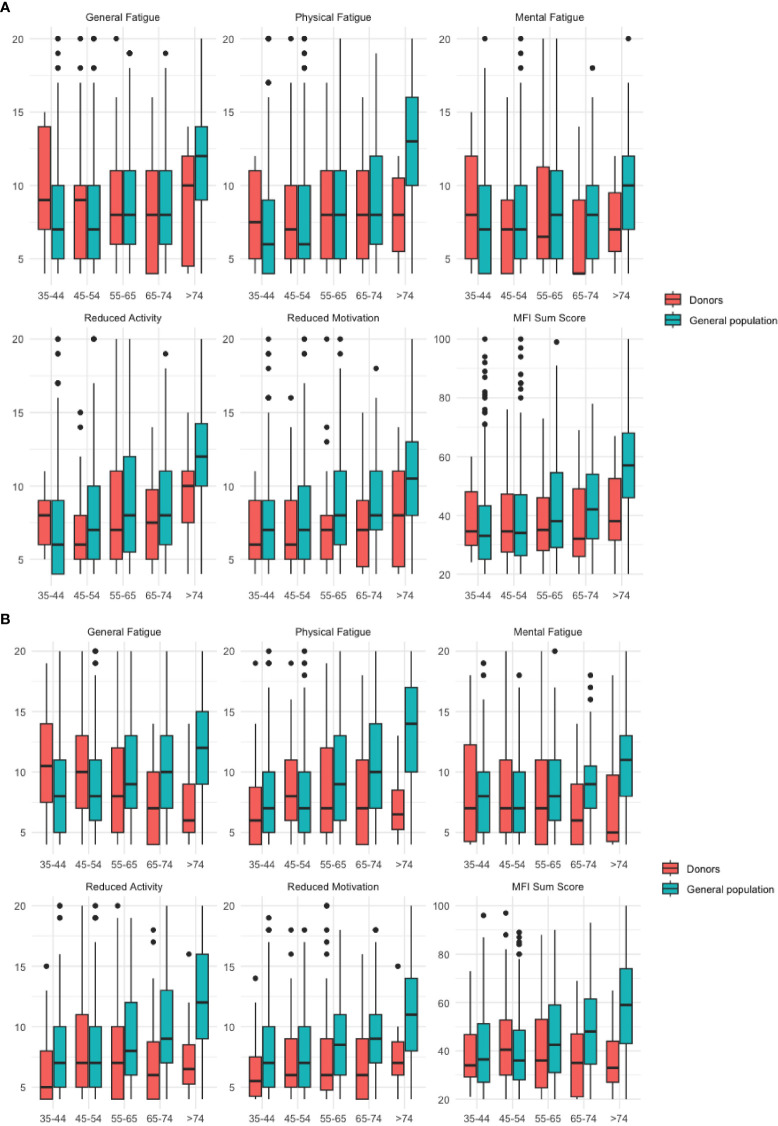
**(A)** Male Subjects. **(B)** Female subjects. Boxplots for the MFI-20 subscales according to age and sex for the donor sample and the general population sample. Significant comparisons are listed in **Supplementary Tables S5**, **S7**. Boxplot for each subscale of the MFI-20 by sex and age groups (years). Y-axis shows fatigue scores (subscale range: 4-20). The horizontal line indicates the median of the distribution, the box represents the lower to upper quartile of the data, the whiskers extend to the last data point over 1.5 times of the interquartile range, circles represent outliers outside of this range).

Fatigue scores of the entire samples and stratified by sex and age are presented in **Supplementary Tables S4**–**S7**. **Figures 1A, B** provides additional information on the descriptive distribution of fatigue scores. Overall, the fatigue scores of the LKD were significantly lower in all MFI-20 subscales in the MFI-20 sum score compared to the population sample except for “general fatigue”. These differences were particularly pronounced in the age group 65-74 and above in both sexes. In younger age groups we did not find significant differences between LKD and the population sample. One exception is the group of 45–54-year-old female donors, who demonstrated significantly higher “general fatigue” scores (p < 0.001) compared to the corresponding comparison group. The effect sizes according to Cohen’s d correspond to moderate to strong effects (**Supplementary Table S7**).

### 3.3 Donors with scores two standard deviations above general population sample means

**Table 2** provides an overview of the number of participants who were two standard deviations above the population mean in the respective sex category. Descriptively, the findings suggest a notable disparity between male and female sex, especially in the MFI-20 subscales “reduced activity” and “mental fatigue” and the MFI-20 sum score.

**Table 2:** Donors 2 SD above the general population sample mean, by sex.

	Male, N (%)	Female, N (%)
General Fatigue	5 (4.13%)	13 (6.34%)
Physical Fatigue	3 (2.48%)	8 (3.9%)
Reduced Activity	1 (0.83%)	11 (5.37%)
Reduced Motivation	2 (1.65%)	8 (3.9%)
Mental Fatigue	6 (4.96%)	19 (9.27%)
MFI-20 Sum score	1 (0.83%)	10 (4.88%)

Absolute number of donors who were 2 SD above the general population. Male donors n = 121 (7 missings), female donors n = 205 (25 missings).

### 3.4 Within group analyses

In the general population sample (**Table 3**), female sex and higher age were significantly associated with higher fatigue scores, whereas higher educated subjects showed significantly lower fatigue scores in most subscales. Adjusted R^2^ ranged from 0.03 to 0.14 for the models. The highest explanatory scores were found for “physical fatigue”, “reduced activity” and the MFI-20 sum score. According to Cohen (24), these scores correspond overall to a moderately explained variance.

**Table 3:** Regression coefficients (with standard error of estimate in parentheses) from the multiple linear regression analysis for the general population sample.

	General Fatigue	Physical Fatigue	Reduced Activity	Reduced Motivation	Mental Fatigue	MFI Sum Score
Intercept	4.51*** (0.41)	2.05*** (0.44)	3.21*** (0.42)	4.76*** (0.36)	5.76*** (0.37)	20.32*** (1.80)
Sex (female)	0.78*** (0.17)	0.58** (0.19)	0.24 (0.18)	0.20 (0.16)	0.44** (0.16)	2.26** (0.78)
Age	0.08*** (0.01)	0.12*** (0.01)	0.10*** (0.01)	0.07*** (0.01)	0.04*** (0.01)	0.40*** (0.03)
Education (A-level)	-0.39 (0.22)	-0.63** (0.23)	-0.68** (0.22)	-0.81*** (0.19)	-0.54** (0.20)	-3.07** (0.96)
adjusted R²	0.08	0.14	0.11	0.08	0.03	0.10
n	1894	1892	1891	1892	1893	1891

Coefficients with *p* < 0.05 in bold.

***p<0.001; **p<0.01; *p<0.05.

In the LKD sample (**Table 4**), age had a negative association with the subscales “general fatigue” and “mental fatigue”. Years since donation was positively associated with the subscale “physical fatigue”. However, the explained variance of the multiple regression was weak (24) with adjusted R^2^ scores ranging from -0.00 to 0.05. The calculation of the interaction effects between age and time since donation did not yield statistically significant results for any of the MFI-20 subscales (all p > 0.05) (**Supplementary Table S9**).

**Table 4:** Regression coefficients (with standard error of estimate in parentheses) from the multiple linear regression analysis for the donor sample.

	General Fatigue	Physical Fatigue	Reduced Activity	Reduced Motivation	Mental Fatigue	MFI Sum Score
Intercept	14.36*** (1.43)	9.50*** (1.35)	7.42*** (1.32)	6.52*** (1.14)	10.21*** (1.38)	44.93*** (5.81)
Sex (female)	0.53 (0.46)	0.06 (0.44)	0.27 (0.43)	0.04 (0.37)	0.29 (0.45)	2.14 (1.87)
Age	-0.10*** (0.02)	-0.03 (0.02)	-0.00 (0.02)	0.01 (0.02)	-0.05* (0.02)	-0.14 (0.10)
Education (A-level)	-0.95 (0.50)	-0.50 (0.48)	-0.44 (0.47)	-0.58 (0.40)	-0.78 (0.49)	-2.69 (2.03)
Years since donation	0.04 (0.04)	0.08* (0.04)	0.05 (0.04)	0.06 (0.03)	0.03 (0.04)	0.32 (0.16)
adjusted R-squared	0.05	0.01	-0.00	0.01	0.01	0.01
n	342	345	343	348	349	325

Coefficients with *p* < 0.05 in bold.

***p<0.001; **p<0.01; *p<0.05.

There was no relationship between fatigue levels and current partnership, recipient group, having felt pressured to donate, and difficulty in the decision to donate. Significant associations were found with regret of donation, relationship and change in relationship with the recipient, perceived health of the recipient, financial situation, and perceived family support after donation (**Table 5**).

**Table 5:** Associations of fatigue scores with donor specific questions.

	General Fatigue		Physical Fatigue		Reduced Activity		Reduced Motivation		Mental Fatigue		MFI Sum Score	
	N	Scores (SD)	N	Scores (SD)	N	Scores (SD)	N	Scores (SD)	N	Scores (SD)	N	Scores (SD)
Regret donation Not at all At least some regret	263 77	8.5 (4.0)*** 11.0 (4.1)	266 77	7.7 (3.6)*** 10.2 (4.3)	262 79	7.2 (3.5)*** 9.8 (4.1)	266 80	6.8 (3.1)*** 8.9 (3.6)	265 82	7.2 (3.7)*** 9.6 (4.4)	251 72	37.5 (15.0)*** 49.0 (18.0)
Financial situation Mainly negative Unchanged Mainly positive	29 283 26	13.6 (4.4)*** 8.6 (3.9) 9.3 (4.2)	29 286 26	12.3 (4.3)*** 7.9 (3.7) 7.7 (3.1)	29 283 27	11.0 (4.6)*** 7.5 (3.6) 7.4 (3.3)	31 286 27	11.0 (4.2)*** 6.9 (3.1) 6.9 (2.2)	31 287 27	10.7 (4.5)*** 7.5 (3.9) 7.3 (3.7)	27 269 25	57.5 (16.9)*** 38.4 (15.6) 37.5 (13.1)
Relationship quality Mainly negative Neutral Mainly positive	5 21 293	11.4 (3.8) 10.8 (4.8) 8.8 (4.0)	4 21 297	10.8 (3.5)* 9.8 (4.3) 8.0 (3.7)	5 21 296	7.8 (3.8) 10.0 (5.2) 7.5 (3.4)	5 21 298	8.6 (2.5)* 8.8 (3.7) 7.0 (3.1)	5 21 299	12.2 (3.1)** 9.3 (4.4) 7.5 (3.8)	4 21 279	48.8 (15.8)* 48.7 (19.6) 38.7 (15.0)
Change in relationship quality Mainly negative Unchanged Mainly positive	18 253 63	12.7 (5.5)* 9.0 (4.0) 8.8 (4.1)	18 255 63	11.6 (5.6)* 8.1 (3.8) 8.1 (3.4)	17 254 64	11.5 (5.7)** 7.4 (3.5) 7.9 (3.7)	18 257 64	10.2 (4.3)** 7.0 (3.1) 7.5 (3.4)	18 259 63	10.7 (4.9)* 7.6 (3.9) 7.7 (3.8)	17 239 61	55.4 (23.8)* 39.0 (15.5) 39.2 (14.4)
Perceived health of recipient Mainly negative Neutral Mainly positive	45 72 198	10.4 (3.9)*** 10.2 (4.2) 8.3 (4.0)	46 75 198	9.6 (4.0)*** 9.2 (3.9) 7.5 (3.6)	44 76 197	8.2 (3.4)*** 9.0 (3.8) 6.9 (3.3)	45 74 202	8.6 (3.7)*** 7.8 (3.3) 6.6 (2.7)	45 76 201	9.4 (4.5)*** 8.4 (3.6) 7.0 (3.7)	39 71 189	47.0 (15.5)*** 44.5 (15.4) 35.9 (14.7)
Reaction of family after donation Mainly negative Neutral Mainly positive	9 31 297	13.0 (4.7)* 8.9 (4.6) 9.0 (4.0)	9 31 300	10.4 (4.4) 8.6 (4.6) 8.2 (3.7)	9 30 299	11.4 (4.3) 7.6 (3.7) 7.2 (3.2)	9 31 304	10.4 (4.3)* 8.4 (4.7) 7.6 (3.9)	9 31 304	10.9 (4.9) 8.4 (4.7) 7.6 (3.9)	9 30 282	56.2 (21.6)* 41.8 (20.6) 39.2 (15.4)

Non-parametric tests (Mann-Whitney-U-Tests, Kruskal-Wallis-Tests); *** <0.001; ** <0.01; * <0.05.

## 4 Discussion

Overall, LKD experienced lower fatigue levels compared to a general population sample. This was particularly evident in older age groups (> 65 years), as LKD maintained stable levels of fatigue while the general population sample tended to experience increasing fatigue. One exception was that female LKD in the age group of 45-54 years showed significantly higher general fatigue scores than the respective age group in the general population sample. In the general population sample, age, gender, and education were significantly associated with fatigue levels, particularly in terms of physical fatigue, reduced activity, and the MFI-20 sum score. However, this did not apply to LKD, even when considering the time since donation and an interaction between age and years since donation.

With a mean time of 7.67 years post-donation, the present study showed mostly lower fatigue scores of donors compared to the respective sex and age group of the general German population. This is not surprising as LKD are a highly selected group and these results are in line with several previous studies. LKD candidates undergo comprehensive medical and psychological evaluation which leads to the exclusion of potential donors with limited health screening before donation (4, 10, 21, 25–27). A Dutch, ten years follow-up study (n = 74) revealed, independent of subgroup analysis, that LKD reported higher MFI-20 scores postoperatively compared to pre-donation scores, except for mental fatigue. However, despite these increased fatigue scores, living donors still exhibited lower fatigue scores compared to the general population sample after ten years (13). Similarly, Rodrigues et al. (6) reported in their longitudinal study comprising 189 living kidney donors with 19 health controls that although LKD suffered significantly more from fatigue than healthy controls one month after donor nephrectomy, no differences in fatigue scores were observed at later time points. Other studies investigating health-related variables using the SF-36 questionnaire also conclude that LKD exhibit no different (7, 28, 29) or even slightly lower (21, 30) scores than the control group.

In line with our findings, Sommerer and colleagues (9) reported in their study with (n = 211) LKD for the age group of > 60-year-old female and male donors significantly lower scores in almost all MFI-20 subscales compared to the German general population. Most likely, older LKD are particularly healthy individuals when they are selected for donation. Minnee et al. (12) found that donors over the age of 60 years showed an advantage in quality of life (QoL; SF-36) compared to donors under the age of 60 years. The authors suggested that older donors do not face their disabilities as quickly as younger donors because they do not have a family to care for or a day job. They also suggest that retirement may have a positive effect on recovery, as retired donors have more time to plan and carry out daily activities and, therefore, experience fewer limitations (12). Additionally, older LKD might be more accustomed to adapt to physical changes and a potential decline in physical capacity, which is an inevitable part of growing older.

It has been suggested that comparisons with the general population sample underestimate possible impairments experienced by living donors (31). Since only candidates with good physical and mental health are eligible for donation, it can be assumed that living donors constitute a selected sample with a generally higher quality of life than the general population. It has been shown that pre-donation QoL scores are higher than that of healthy individuals. Following this reasoning and previous research, below average fatigue levels can be expected in LKD pre-donation (10). However, it cannot be excluded that LKD candidates might (consciously or unconsciously) overstate their preoperative QoL and understate their preoperative fatigue to not be rejected as living donors (15). Prospective studies demonstrated an increase in fatigue lasting up to 24 months or even longer in some LKD (6, 10, 11). At the same time, risk factors having the potential to negatively impact fatigue were only recorded to a limited extent in previous longitudinal trials and the clinical significance of the observed changes is still unknown.

Interestingly, our MFI-20 scores are similar to the data reported by Suwelack (10) one year after donation and by Sommerer (9) almost 10 years after donation (**Tables 6A, B**). While the study by Sommerer et al. (9) - as our study - was not prospective, Suwelack et al. (10) reported pre-donation scores and found an increase specifically in mental fatigue with values that were below population norms pre-donation and above population norms 1-year after donation. The number of LKD exceeding 1 SD of population norms in our sample closely resemble those in the study by Suwelack et al. (10) (**Table 6A**). Taken together, when combining the results of other German studies, it appears that even though LKD have comparable or even lower fatigue scores compared to general population samples, some might still experience a significant and clinically relevant increase of fatigue post-donation. Post-donation fatigue levels in LKD that are above the population norms are of particular concern. Descriptive statistics of individual comparisons in our study showed, that there were donors who even exceeded the cutoff of two standard deviations of the population mean of the respective sex and age category, suggesting that these donors experienced a level of fatigue that is most likely clinically relevant. If this is associated with the kidney donation can, however, not be determined. Even though, the number of LKD in this fatigue level is small, from a clinical point of view, it is of utmost importance to identify the donors with clinically elevated fatigue scores.

**Table 6A:** Comparisons with German studies using the MFI-20.

	Suwelack et al., 2022 Pre-donation		Suwelack et al., 2022 1-year post-donation	Our sample 7.7 years post-donation	
	n	Median (25%-75% percentile)			n*
General Fatigue	255	7.00 (4-9)	8.00 (5-11)	8.00 (5-12)	343
Physical Fatigue	252	6.00 (5-9)	7.00 (5-9.83)	8.00 (5-11)	346
Reduced Activity	231	6.00 (5-8)	7.00 (5-9)	7.00 (5-10)	344
Reduced Motivation	230	6.50 (5-8)	6.00 (5-8)	6.00 (5-9)	349
Mental Fatigue	245	6.00 (4-8)	9.00 (8-11)	7.00 (4-10)	350
	Clinically relevant fatigue (1SD above population norms) in %					
General Fatigue (>12.2)	6.3%		18.4%	20.7%	
Mental Fatigue (>10.9)	10.6%		28.1%	23.4%	

*Without age groups ≤ 24, 25-34.

**Table 6B:** Comparison with German studies using the MFI-20.

	Sommerer et al., 2018* 9.7 years post donation		Our sample 7.7 years post donation	
	n	Mean (SD)		n**
General Fatigue	209	9.35 (4.51)	9.09 (4.18)	343
Physical Fatigue	210	8.35 (4.56)	8.27 (3.87)	346
Reduced Activity	211	8.11 (3.89)	7.75 (3.77)	344
Reduced Motivation	211	7.50 (3.58)	7.30 (3.31)	349
Mental Fatigue	211	7.88 (3.75)	7.80 (4.00)	350

*Values were calculated from Sommerer et al., 2018.

**Without age groups ≤ 24, 25-34.

Female donors in the age group of 45-54 years were the only subgroup in this study that showed significantly higher fatigue scores than the general population sample in the subscale “general fatigue”. These results are partly in line with the results of Sommerer et al. (9), who also reported significantly higher fatigue scores in middle-aged female donors (40-59 years) in the subscales of general fatigue, but also physical fatigue.

One possible explanation for these findings could be physiological changes associated with the menopause, which are common in women of this age group. It is known that hormonal fluctuations during menopause, such as changes in estrogen and progesterone levels, generally disrupt sleep behavior and lead to lower energy levels (32, 33), which could contribute to fatigue and may influence the physical adaptation to nephrectomy in this donor group. Additionally, women in this age group are often faced with dual responsibilities, such as childcare and caring for elderly relatives, which can lead to increased stress and fatigue (9). Nevertheless, as women in the general population sample also experience menopause, including the physiological changes associated with it, it can only be speculated that there could be specific mechanisms enhancing the effects of menopause in women with only one kidney. However, as far as we know, so far there has been no research examining this theory.

The results of the multiple regression for the general population sample showed, with few exceptions, a significant positive association between fatigue levels and female sex and higher age, and a significant negative association of education. These findings confirm previous studies. A study in a representative sample of the Swedish general population sample confirms the sex difference, reporting significantly higher fatigue scores for women than for men on all MFI-20 subscales (34). Watt et al. (35) found slight sex differences with generally higher mean fatigue scores in women in a Danish general population sample. Some further studies report more fatigue in women than in men, and an almost linear increase with age (20, 22, 36). The positive effect of higher education on reduced fatigue scores seems to be well supported by previous research (34, 35).

In comparison to the general population sample, multiple linear regression analysis in the donor sample revealed virtually no association between fatigue and sex, age, and education level. Also, years since donation was unrelated to fatigue. The interaction effect between age and time since donation was also non-significant, indicating that the relationship between years since donation and fatigue is independent of age. Furthermore, the explanatory power of the models was generally low and other variables than age, sex, or education seem to play a role in the donor population compared to the general population.

In the literature, inconclusive results have been reported regarding associations of socioeconomic variables among LKD. For example, the presented findings are in contrast to a positive linear relationship between age and fatigue reported by Wirken et al. (8). However, the present findings are consistent with several other studies that have not found a significant relationship between age and fatigue (4, 9). Regarding sex, some studies also report an association between sex and fatigue in the donor population, with higher fatigue scores in women (4, 16), while other studies could not clearly show this association (6, 9).

Our results reaffirm that most LKD were satisfied with their decision to donate, did not feel pressured, and had a positive relationship with the recipient. Nevertheless, our results concerning the association between fatigue levels and donor specific factors are mostly in line with the existing literature. LKD who had experienced a negative change in the relationship with the recipient, lower appreciation from their family, and adverse outcomes for the recipient have been repeatedly found to be associated with adverse psychosocial outcome for LKD in previous studies (4, 37–40). However, we cannot simply assume causation in one direction. The majority of LKD did not regret donation and would be willing to donate again; still, LKD with at least some regret reported higher fatigue scores (4). In the U.S. an association between financial burdens related to donation and lower satisfaction with life has been reported (39). Again, we cannot differentiate between cause and consequence. Enhanced post-donation monitoring and attention to donors at greater psychosocial risk may improve long-term donor outcomes (40).

### 4.1 Strengths and limitations of the study

The strength of the present study is the sample size with a fairly high response rate 7 years after donation. The dataset also had few missing data. We analyzed the data by age and sex strata which gave new insights into subgroups. The use of the previously validated MFI-20 questionnaire in a representative sample of the German population, is a further strength of this study allowing the accurate assessment of self-reported fatigue (20).

Limitations are the lack of pre-donation data and the use of a population sample as control group. Regarding the cross-sectional design of this study, baseline data would be preferable to allow further statements about fatigue development over time in the donor sample. Therefore, it is not possible to draw causal conclusions about the association between donation and fatigue in this study. However, longitudinal studies are often expensive and associated with high drop-out rates.

If a healthy comparison group of potential donors would be used, the size of this comparison group might be limited, making it challenging to statistically detect smaller differences or associations. On the other hand, one advantage of using the general population sample as a broader comparison group is that it can provide thresholds that help identify clinically relevant fatigue in donors. Comparing LKD with a general population sample inevitably leads to unequal sample sizes. Subgroup comparisons are often faced with very small sample sizes, which was also the challenge in this study. However, the relatively large sample size of LKD in the present study enabled many age and sex groups to be compared.

Limitations of this study also include the disparity in the time points at which the data was collected. The fatigue date of the living kidney donors (LKD) in our study ranged from 2016 to 2017, while the data of the general population sample was collected in 2021. The time span between the data collection periods could potentially introduce confounding factors such as changes in medical practices, i. e. improvements in preoperative evaluations or postoperative care, which may have influenced the outcomes. Additional limitations concern the non-response rate of over 40% among the donors which introduces the potential for a selection bias (17, 18). As the data on living kidney donation is a single-center study, generalizations of these results should be interpreted carefully. A further limitation of this study is the underrepresentation of younger donors, particularly those in the two youngest age groups (under 24 and 25-34 years). The low representation of younger donors is also seen in the German sample of Sommerer et al. (9), with n = 2 for the age category 25-34 and n = 10 for the age category 35-44. This circumstance may result from the strict German transplant act on living organ donation as well as from some doctors’ reluctance e.g., to allow young women who still wish to have children to donate a kidney due to potentially elevated risks during pregnancy.

## 5 Conclusion

Based on the large donor sample, the current findings provide new information to the research gap in the age- and sex-related comparison of fatigue scores between LKD and the general population, indicating that donors mostly show lower fatigue scores 7.67 years post donation than the general German population. Our results suggest that middle-aged female donors may require more frequent monitoring and possibly further medical support postoperatively as they turn out to be the only donor subgroup in our study with significantly higher post-donation fatigue levels than the corresponding subgroup in the general population. Nevertheless, it needs to be acknowledged that there are individuals who showed high fatigue levels (2 SD above norm scores). There is a need for future research regarding the assessment of clinical relevance of fatigue by validated threshold scores (10). The examined sociodemographic variables, which are well established in explaining fatigue levels in the general population sample do almost not reveal any explanatory contribution in the donor sample. Donor specific factors may contribute to these higher fatigue scores such as the relationship with the recipient, perceived recipient health, or financial burden. Therefore, further studies should prospectively investigate for donor specific factors associated with fatigue after kidney donation to gain a deeper understanding of their impact. Additionally, the identification and evaluation of suitable control groups could be helpful to better understand the results of LKD samples.

## Data Availability

The raw data supporting the conclusions of this article will be made available by the authors, without undue reservation.

## References

[1] Deutsche Stiftung Organtransplantation. Nierentransplantation nach Lebendspende. Available online at: https://www.dso.de/DSO-Infografiken/NierenTX_nach%20Lebendspende.png (Accessed December 23, 2024).

[2] Gesetz über die Spende, Entnahme und Übertragung von Organen und Geweben (Transplantationsgesetz - TPG) (1997/2003). Available online at: https://www.gesetze-im-internet.de/tpg/ (Accessed December 23, 2024).

[3] Horvat LD Shariff SZ Garg AX. Donor Nephrectomy Outcomes Research (DONOR) Network. Global trends in the rates of living kidney donation. Kidney Int. (2009) 75:1088–98. doi: 10.1038/ki.2009.20 19225540

[4] Meyer K Wahl AK Bjørk IT Wisløff T Hartmann A Andersen MH. Long-term, self-reported hea3lth outcomes in kidney donors. BMC Nephrol. (2016) 17:8. doi: 10.1186/s12882-016-0221-y 26754798 PMC4709885

[5] Janki S Klop KWJ Hagen SM Terkivatan T Betjes MGH Tran TCK. Robotic surgery rapidly and successfully implemented in a high volume laparoscopic center on living kidney donation. Int J Med Robot. (2017) 13. doi: 10.1002/rcs.1743 26987773

[6] Rodrigue JR Fleishman A Schold JD Morrissey P Whiting J Vella J. Patterns and predictors of fatigue following living donor nephrectomy: Findings from the KDOC Study. Am J Transplant. (2020) 20:181–9. doi: 10.1111/ajt.15519 31265199

[7] Wirken L van Middendorp H Hooghof CW Rovers MM Hoitsma AJ Hilbrands LB. The course and predictors of health-related quality of life in living kidney donors: A systematic review and meta-analysis. Am J Transplant. (2015) 15:3041–54. doi: 10.1111/ajt.13453 26414703

[8] Wirken L van Middendorp H Hooghof CW Sanders JF Dam RE van der Pant KAMI. Psychosocial consequences of living kidney donation: a prospective multicentre study on health-related quality of life, donor-recipient relationships and regret. Nephrol Dial Transplant. (2019) 34:1045–55. doi: 10.1093/ndt/gfy307 30544241

[9] Sommerer C Estelmann S Metzendorf NG Leuschner M Zeier M. Gender disparity in health-related quality of life and fatigue after living renal donation. BMC Nephrol. (2018) 19:377. doi: 10.1186/s12882-018-1187-8 30587146 PMC6307222

[10] Suwelack B Berger K Wolters H Gerß JWO Bormann E Wörmann V. Results of the prospective multicenter SoLKiD cohort study indicate bio-psycho-social outcome risks to kidney donors 12 months after donation. Kidney Int. (2022) 101:597–606. doi: 10.1016/j.kint.2021.12.007 34953772

[11] Dols LF Ijzermans JN Wentink N Tran TC Zuidema WC Dooper IM. Long-term follow-up of a randomized trial comparing laparoscopic and mini-incision open live donor nephrectomy. Am J Transplant. (2010) 10:2481–7. doi: 10.1111/j.1600-6143.2010.03281.x 20977639

[12] Minnee RC Bemelman WA Polle SW van Koperen PJ Ter Meulen S Donselaar-van der Pant KA. Older living kidney donors: surgical outcome and quality of life. Transplantation. (2008) 86:251–6. doi: 10.1097/TP.0b013e31817789dd 18645487

[13] Janki S Klop KW Dooper IM Weimar W Ijzermans JN Kok NF. More than a decade after live donor nephrectomy: a prospective cohort study. Transpl Int. (2015) 28:1268–75. doi: 10.1111/tri.12589 25865340

[14] Kok NF Alwayn IP Tran KT Hop WC Weimar W Ijzermans JN. Psychosocial and physical impairment after mini-incision open and laparoscopic donor nephrectomy: A prospective study. Transplantation. (2006) 82:1291–7. doi: 10.1097/01.tp.0000239312.45050.05 17130777

[15] Kroencke S Nashan B Fischer L Erim Y Schulz KH. Donor quality of life up to two years after living donor liver transplantation: a prospective study. Transplantation. (2014) 97:582–9. doi: 10.1097/01.TP.0000438206.04348.b2 24595117

[16] Meyer KB Hartmann A Mjøen G Andersen MH. Relationships between clinical, self-reported, and donation specific outcomes: A prospective follow-up study 10 years after kidney donation. Ann Transplant. (2017) 22:148–55. doi: 10.12659/aot.902330 PMC12577502 28321111

[17] Nöhre M Pollmann I Mikuteit M Weissenborn K Gueler F. de Zwaan M. Partnership satisfaction in living kidney donors. Front Psychiatry. (2018) 9:353. doi: 10.3389/fpsyt.2018.00353 30123146 PMC6085414

[18] Pollmann I Gueler F Mikuteit M Nöhre M Richter N Weissenborn K. Adaptive personality traits and psychosocial correlates among living kidney donors. Front Psychiatry. (2017) 8:210. doi: 10.3389/fpsyt.2017.00210 29109691 PMC5660284

[19] Smets EM Garssen B Bonke B De Haes JC. The Multidimensional Fatigue Inventory (MFI) psychometric qualities of an instrument to assess fatigue. J Psychosom Res. (1995) 39:315–25. doi: 10.1016/0022-3999(94)00125-o 7636775

[20] Westenberger A Nöhre M Brähler E Morfeld M de Zwaan M. Psychometric properties, factor structure, and German population norms of the multidimensional fatigue inventory (MFI-20). Front Psychiatry. (2022) 13:1062426. doi: 10.3389/fpsyt.2022.1062426 36606126 PMC9807811

[21] De Groot IB Stiggelbout AM van der Boog PJ Baranski AG Marang-van de Mheen PJ PARTNER-study group. Reduced quality of life in living kidney donors: association with fatigue, societal participation and pre-donation variables. Transpl Int. (2012) 25:967–75. doi: 10.1111/j.1432-2277.2012.01524.x 22780196

[22] Schwarz R Krauss O Hinz A. Fatigue in the general population. Onkologie. (2003) 26:140–4. doi: 10.1159/000069834 12771522

[23] Krauß O. MFI-20. Multidimensional fatigue inventory. In: Schumacher J Klaiberg A Brähler E, editors. Diagnostische Verfahren zu Lebensqualität und Wohlbefinden. Hogrefe, Göttingen (2003). p. 216–9.

[24] Cohen J. Statistical Power Analysis for the Behavioral Sciences. 2nd ed. New York: Routledge. (1988). doi: 10.4324/9780203771587

[25] Biller-Andorno N. Gender imbalance in living organ donation. Med Health Care Philos. (2002) 5:199–204. doi: 10.1023/a:1016053024671 12168995

[26] Leifeld S de Zwaan M Albayrak Ö Einecke G Nöhre M. Live Donor Assessment Tool (LDAT): Reliability and validity of the German version in living kidney donor candidates. J Acad Consult Liaison Psychiatry. (2023) 64:429–35. doi: 10.1016/j.jaclp.2023.03.002 36963466

[27] Klop KW Dols LF Weimar W Dooper IM IJzermans JN Kok NF. Quality of life of elderly live kidney donors. Transplantation. (2013) 96:644–8. doi: 10.1097/TP.0b013e31829e6d9b 23860088

[28] Clemens K Boudville N Dew MA Geddes C Gill JS Jassal V. The long-term quality of life of living kidney donors: a multicenter cohort study. Am J Transplant. (2011) 11:463–9. doi: 10.1111/j.1600-6143.2010.03424.x 21342446

[29] Maglakelidze N Pantsulaia T Managadze L Chkhotua A. Assessment of health-related quality of life in living kidney donors. Transplant Proc. (2011) 43:373–5. doi: 10.1016/j.transproceed.2010.12.016 21335225

[30] Isotani S Fujisawa M Ichikawa Y Ishimura T Matsumoto O Hamami G. Quality of life of living kidney donors: the short-form 36-item health questionnaire survey. Urology. (2002) 60:588–92. doi: 10.1016/s0090-4295(02)01865-4 12385912

[31] Suwelack B Nöhre M. Lebendnierenspende - Muss das Risiko neu bewertet werden? Die Nephrologie. (2024) 19:95–101. doi: 10.1007/s11560-023-00705-y

[32] El Khoudary SR Greendale G Crawford SL Avis NE Brooks MM Thurston RC. The menopause transition and women's health at midlife: a progress report from the Study of Women's Health Across the Nation (SWAN). Menopause. (2019) 26:1213–27. doi: 10.1097/GME.0000000000001424 PMC6784846 31568098

[33] Lu J Li K Zheng X Liu R Chen M Xian J. Prevalence of menopausal symptoms and attitudes towards menopausal hormone therapy in women aged 40-60 years: a cross-sectional study. BMC Womens Health. (2023) 23:472. doi: 10.1186/s12905-023-02621-8 37667324 PMC10476428

[34] Engberg I Segerstedt J Waller G Wennberg P Eliasson M. Fatigue in the general population- associations to age, sex, socioeconomic status, physical activity, sitting time and self-rated health: the northern Sweden MONICA study 2014. BMC Public Health. (2017) 17:654. doi: 10.1186/s12889-017-4623-y 28806984 PMC5557471

[35] Watt T Groenvold M Bjorner JB Noerholm V Rasmussen NA Bech P. Fatigue in the Danish general population. Influence of sociodemographic factors and disease. J Epidemiol Community Health. (2000) 54:827–33. doi: 10.1136/jech.54.11.827 PMC1731588 11027196

[36] Hinz A Barboza CF Barradas S Körner A Beierlein V Singer S. Fatigue in the general population of Colombia - normative values for the multidimensional fatigue inventory MFI-20. Onkologie. (2013) 36:403–7. doi: 10.1159/000353606 23921758

[37] Watson JM Behnke MK Fabrizio MD McCune TR. Recipient graft failure or death impact on living kidney donor quality of life based on the living organ donor network database. J Endourol. (2013) 27:1525–9. doi: 10.1089/end.2013.0189 24134317

[38] Tong A Chapman JR Wong G Kanellis J McCarthy G Craig JC. The motivations and experiences of living kidney donors: a thematic synthesis. Am J Kidney Dis. (2012) 60:15–26. doi: 10.1053/j.ajkd.2011.11.043 22305757

[39] Jacobs CL Gross CR Messersmith EE Hong BA Gillespie BW Hill-Callahan P. Emotional and financial experiences of kidney donors over the past 50 years: the RELIVE study. Clin J Am Soc Nephrol. (2015) 10:2221–31. doi: 10.2215/CJN.07120714 PMC4670771 26463883

[40] Massey EK Rule AD Matas AJ. Living kidney donation: A narrative review of mid- and long-term psychosocial outcomes. Transplantation. (2024). doi: 10.1097/TP.0000000000005094 PMC11652709 38886889

