# Supplementary material for: Fatigue in living kidney donors compared to a German general population sample: an exploratory study
Source: Front Psychiatry. 2025 Jan 30;15:1510738. doi: 10.3389/fpsyt.2024.1510738 (PMC11821954; doi:10.3389/fpsyt.2024.1510738)
Supplement: Supplementary file 1 [file Table1.docx]

Supplementary Material

**Table S1:** Subgroup formation for the MFI-20 subscale score comparisons

| Age groups | Population sample | Donor sample |
| --- | --- | --- |
| ≤24 | 229 | 0 |
| 25-34 | 383 | 3 |
| 35-44 | 392 | 23 |
| 45-54 | 464 | 113 |
| 55-64 | 480 | 122 |
| 65-74 | 341 | 78 |
| >74 | 220 | 22 |

**Table S2:** Absolute response frequencies for the MFI-20 subscale score comparisons

| MFI-20 | Population sample | Donor sample |
| --- | --- | --- |
| General Fatigue | 1894 | 343 |
| Physical Fatigue | 1892 | 346 |
| Reduced Activity | 1891 | 344 |
| Reduced Motivation | 1892 | 349 |
| Mental Fatigue | 1893 | 350 |
| MFI-20 Sum score | 1891 | 326 |
| All items answered in the subscales, without participants < 35 years in the donor sample and without individuals identifying as diverse sex in the general population. When comparing sum scores, only subjects who completed all subscales were included. | | |

| **Table S3:** Donor/recipient relationships in the different donor age strata. In parentheses, percentages within the subgroups. | | | | | | | | | | | |
| --- | --- | --- | --- | --- | --- | --- | --- | --- | --- | --- | --- |
| Age group/ Sex | n | Years since donation (mean) | Spouse | Mother/ Father | Daughter/ Son | Brother/ Sister | Aunt/ Uncle/ Cousin | Brother-in-law/ Sister-in-law/ Son-in-law | Boyfriend/ Girlfriend | Grandson/ Grand-daughter | Nephew/ Niece |
|  |  |  |  |  |  |  |  |  |  |  |  |
| 25-34  M | 2 | 3.50 |  |  | 1 (50) | 1 (50) |  |  |  |  |  |
| 25-34  F | 1 | 1 |  |  | 1 (100) |  |  |  |  |  |  |
| 35-44  M | 8 | 4.63 | 2 (2.05) |  | 2 (25) | 4 (50) |  |  |  |  |  |
| 35-44  F | 15 | 4.87 | 6 (40.0) |  | 4 (26.7) | 3 (20.0) |  |  | 2 (13.3) |  |  |
| 45-54  M | 35 | 7.09 | 10 (28.6) |  | 18 (51.4) | 5 (14.3) |  |  | 1 (2.9) |  | 1 (2.9) |
| 45-54  F | 78 | 6.31 | 37 (47.4) | 1 (1.3) | 28 (35.9) | 7 (9.0) |  | 2 (2.6) |  |  | 3 (3.9) |
| 55-64  M | 48 | 7.35 | 14 (29.2) |  | 21 (43.8) | 7 (14.6) | 1 (2.1) | 3 (6.3) | 2 (4.2) |  |  |
| 55-64  F | 74 | 8.07 | 30 (40.5) |  | 34 (46.0) | 5 (6.8) |  | 2 (2.7) | 1 (1.4) | 1 (1.4) | 1 (1.4) |
| 65-74  M | 29* | 10.03 | 10 (35.7) |  | 17 (60.7) | 1 (3.6) |  |  |  |  |  |
| 65-74  F | 49 | 8.50 | 21 (42.9) |  | 21 (42.9) | 3 (6.1) | 1 (2.0) |  | 1 (2.0) | 1 (2.0) | 1 (2.0) |
| >74  M | 8 | 11.62 | 2 (25) |  | 6 (75) |  |  |  |  |  |  |
| >74  F | 14 | 10.57 | 3 (21.4) |  | 9 (64.3) | 2 (14.3) |  |  |  |  |  |
| F = female, M = male; n = 361, *1 missing score of recipients in the subgroup of 65-74 M | | | | | | | | | | | |

**Table S4**: Comparison of the MFI-20 subscale scores and sum scores between the LKD and the general population sample

| **MFI Scale** | **Group** | **n** | **M** | **SD** | **Min** | **q25** | **Median** | **q75** | **Max** |
| --- | --- | --- | --- | --- | --- | --- | --- | --- | --- |
| General Fatigue | population | 1894 | 9.12 | 3.95 | 4 | 6 | 8 | 12 | 20 |
|  | LKD | 343 | 9.09 | 4.18 | 4 | 5 | 8 | 12 | 20 |
| Physical Fatigue | population | 1892 | 9.08 | 4.4 | 4 | 5 | 8 | 12 | 20 |
|  | LKD | 346 | 8.27 | 3.87 | 4 | 5 | 8 | 11 | 20 |
| Reduced Activity | population | 1891 | 8.8 | 4.11 | 4 | 5 | 8 | 12 | 20 |
|  | LKD | 344 | 7.75 | 3.77 | 4 | 5 | 7 | 10 | 20 |
| Reduced Motivation | population | 1892 | 8.54 | 3.52 | 4 | 6 | 8 | 11 | 20 |
|  | LKD | 349 | 7.3 | 3.31 | 4 | 5 | 6 | 9 | 20 |
| Mental Fatigue | population | 1893 | 8.31 | 3.51 | 4 | 5 | 8 | 11 | 20 |
|  | LKD | 350 | 7.8 | 4 | 4 | 4 | 7 | 10 | 20 |
| MFI-20 Sumscore | population | 1891 | 43.86 | 17.82 | 20 | 29 | 40 | 57 | 100 |
|  | LKD | 326 | 40.01 | 16.33 | 20 | 27 | 35.5 | 49 | 97 |

M = mean, SD = standard deviation, q25 = lower quartile, q75 = upper quartile

**Table S5**: Statistics and effect sizes for the Wilcoxon-Mann-Whitney U test for the comparison of the MFI-20 subscale scores and sum scores between the LKD and the general population sample

| General Fatigue | W = 329963, p = 0.64, d = 0.0066, 95 % - CI [-0.108, 0.122] |
| --- | --- |
| Physical Fatigue | W = 359489, **p < 0.01**, d = 0.187, 95 % - CI [ 0.0721, 0.302] |
| Reduced Activity | W = 376080, **p < 0.001**, d = 0.259, 95% - CI [ 0.143, 0.374] |
| Reduced Motivation | W = 404577, **p < 0.001**, d = 0.355, 95 % - CI [0.240, 0.470] |
| Mental Fatigue | W = 372677, **p < 0.001**, d = 0.143, 95 % - CI [0.0288, 0.257] |
| MFI Sumscore | W = 346861, **p < 0.001**, d = 0.219, 95 %- CI [0.101, 0.337] |

CI = confidence interval

**Table S6**: Comparisons of the MFI-20 subscale scores and sum scores between the LKD and the general population sample by age and sex

**General Fatigue**

| Group | Sex | n | M | SD | Min | q25 | Median | q75 | Max |
| --- | --- | --- | --- | --- | --- | --- | --- | --- | --- |
| 35-44 | | | | | | | | | |
| population | male | 198 | 7.70 | 3.77 | 4 | 5.0 | 7.0 | 9.00 | 20 |
| donors | male | 8 | 10.00 | 4.69 | 4 | 6.5 | 10.0 | 14.25 | 15 |
| population | female | 193 | 8.83 | 3.96 | 4 | 5.0 | 8.0 | 12.00 | 19 |
| donors | female | 15 | 11.13 | 4.84 | 4 | 7.0 | 10.0 | 14.50 | 19 |
| 45-54 | | | | | | | | | |
| population | male | 218 | 7.95 | 3.58 | 4 | 5.0 | 7.0 | 10.00 | 20 |
| donors | male | 35 | 9.06 | 4.43 | 4 | 5.5 | 9.0 | 11.00 | 20 |
| population | female | 246 | 8.43 | 3.82 | 4 | 5.0 | 7.0 | 11.00 | 20 |
| donors | female | 76 | 10.16 | 4.07 | 4 | 7.0 | 10.0 | 12.00 | 20 |
| 55-64 | | | | | | | | | |
| population | male | 237 | 8.72 | 3.87 | 4 | 6.0 | 8.0 | 11.00 | 20 |
| donors | male | 47 | 8.72 | 3.53 | 4 | 6.0 | 8.0 | 11.00 | 20 |
| population | female | 242 | 9.70 | 3.92 | 4 | 7.0 | 9.0 | 13.00 | 20 |
| donors | female | 71 | 9.55 | 4.63 | 4 | 5.0 | 9.0 | 13.00 | 20 |
| 65-74 | | | | | | | | | |
| population | male | 180 | 9.12 | 3.48 | 4 | 7.0 | 9.0 | 12.00 | 19 |
| donors | male | 28 | 7.89 | 4.00 | 4 | 4.0 | 7.5 | 11.00 | 16 |
| population | female | 160 | 10.01 | 3.99 | 4 | 7.0 | 10.0 | 13.00 | 20 |
| donors | female | 43 | 7.42 | 3.33 | 4 | 4.0 | 7.0 | 10.00 | 14 |
| >74 | | | | | | | | | |
| population | male | 108 | 11.50 | 3.46 | 4 | 9.0 | 12.0 | 14.00 | 20 |
| donors | male | 7 | 8.71 | 4.27 | 4 | 4.5 | 10.0 | 12.00 | 14 |
| population | female | 112 | 11.89 | 3.81 | 4 | 9.0 | 12.0 | 15.00 | 20 |
| donors | female | 13 | 7.15 | 3.05 | 4 | 5.0 | 6.0 | 9.00 | 14 |

M = mean, SD = standard deviation, q25 = lower quartile, q75 = upper quartile

**Physical Fatigue**

| Group | Sex | n | M | SD | Min | q25 | Median | q75 | Max |
| --- | --- | --- | --- | --- | --- | --- | --- | --- | --- |
| 35-44 | | | | | | | | | |
| population | male | 197 | 7.39 | 4.01 | 4 | 4.00 | 6.0 | 9.0 | 20 |
| donors | male | 7 | 7.71 | 3.45 | 4 | 5.00 | 6.0 | 11.0 | 12 |
| population | female | 193 | 7.98 | 3.82 | 4 | 5.00 | 7.0 | 10.0 | 20 |
| donors | female | 15 | 7.80 | 4.43 | 4 | 4.50 | 7.0 | 8.5 | 19 |
| 45-54 | | | | | | | | | |
| population | male | 217 | 7.59 | 3.86 | 4 | 5.00 | 6.0 | 10.0 | 20 |
| donors | male | 35 | 8.46 | 4.36 | 4 | 5.00 | 7.0 | 9.5 | 20 |
| population | female | 246 | 7.83 | 3.79 | 4 | 5.00 | 7.0 | 10.0 | 19 |
| donors | female | 76 | 8.54 | 3.72 | 4 | 5.00 | 8.0 | 11.0 | 19 |
| 55-64 | | | | | | | | | |
| population | male | 237 | 8.57 | 4.27 | 4 | 5.00 | 7.0 | 11.0 | 20 |
| donors | male | 48 | 8.48 | 3.92 | 4 | 5.00 | 8.0 | 11.0 | 17 |
| population | female | 242 | 9.52 | 4.20 | 4 | 6.00 | 8.0 | 13.0 | 20 |
| donors | female | 71 | 8.72 | 4.29 | 4 | 5.00 | 8.0 | 12.0 | 19 |
| 65-74 | | | | | | | | | |
| population | male | 180 | 9.51 | 3.87 | 4 | 6.00 | 9.0 | 12.0 | 19 |
| donors | male | 28 | 7.79 | 3.40 | 4 | 4.75 | 8.0 | 11.0 | 16 |
| population | female | 160 | 10.44 | 4.35 | 4 | 7.00 | 10.0 | 14.0 | 20 |
| donors | female | 45 | 7.69 | 3.67 | 4 | 4.00 | 7.0 | 10.0 | 18 |
| >74 | | | | | | | | | |
| population | male | 108 | 13.06 | 4.08 | 4 | 10.00 | 13.0 | 16.0 | 20 |
| donors | male | 7 | 8.00 | 3.32 | 4 | 5.50 | 8.0 | 10.5 | 12 |
| population | female | 112 | 13.26 | 4.36 | 4 | 10.00 | 14.0 | 17.0 | 20 |
| donors | female | 14 | 7.21 | 2.81 | 4 | 5.25 | 6.5 | 8.5 | 13 |

M = mean, SD = standard deviation, q25 = lower quartile, q75 = upper quartile

**Reduced Activity**

| Group | Sex | n | M | SD | Min | q25 | Median | q75 | Max |
| --- | --- | --- | --- | --- | --- | --- | --- | --- | --- |
| 35-44 | | | | | | | | | |
| population | male | 196 | 7.47 | 3.83 | 4 | 4.00 | 6.0 | 9.00 | 20 |
| donors | male | 8 | 7.88 | 1.89 | 5 | 6.75 | 8.0 | 9.00 | 11 |
| population | female | 193 | 7.97 | 3.64 | 4 | 5.00 | 7.0 | 11.00 | 19 |
| donors | female | 15 | 7.07 | 3.43 | 4 | 4.50 | 5.0 | 8.00 | 15 |
| 45-54 | | | | | | | | | |
| population | male | 218 | 7.75 | 3.72 | 4 | 5.00 | 7.0 | 10.00 | 20 |
| donors | male | 34 | 6.85 | 3.18 | 4 | 4.25 | 5.5 | 8.00 | 15 |
| population | female | 245 | 7.56 | 3.57 | 4 | 5.00 | 7.0 | 10.00 | 20 |
| donors | female | 77 | 8.21 | 4.28 | 4 | 5.00 | 7.0 | 10.00 | 20 |
| 55-64 | | | | | | | | | |
| population | male | 236 | 8.46 | 4.05 | 4 | 5.00 | 7.0 | 11.00 | 20 |
| donors | male | 47 | 7.66 | 2.94 | 4 | 5.00 | 7.0 | 10.00 | 14 |
| population | female | 243 | 8.92 | 4.05 | 4 | 5.00 | 8.0 | 12.00 | 20 |
| donors | female | 71 | 8.28 | 4.24 | 4 | 5.00 | 8.0 | 11.00 | 20 |
| 65-74 | | | | | | | | | |
| population | male | 180 | 9.33 | 3.71 | 4 | 6.00 | 9.0 | 12.00 | 19 |
| donors | male | 29 | 7.83 | 3.96 | 4 | 5.00 | 7.0 | 10.00 | 20 |
| population | female | 160 | 9.81 | 4.04 | 4 | 6.75 | 9.0 | 13.00 | 20 |
| donors | female | 42 | 6.86 | 3.49 | 4 | 4.00 | 5.5 | 8.00 | 18 |
| >74 | | | | | | | | | |
| population | male | 108 | 12.14 | 3.78 | 4 | 10.00 | 12.0 | 14.25 | 20 |
| donors | male | 7 | 9.43 | 3.64 | 4 | 7.50 | 10.0 | 11.00 | 15 |
| population | female | 112 | 12.27 | 4.37 | 4 | 9.00 | 12.0 | 16.00 | 20 |
| donors | female | 14 | 7.43 | 3.48 | 4 | 5.25 | 6.5 | 8.50 | 16 |

M = mean, SD = standard deviation, q25 = lower quartile, q75 = upper quartile

**Reduced Motivation**

| Group | Sex | n | M | SD | Min | q25 | Median | q75 | Max |
| --- | --- | --- | --- | --- | --- | --- | --- | --- | --- |
| 35-44 | | | | | | | | | |
| population | male | 197 | 7.55 | 3.41 | 4 | 5.00 | 7.0 | 9.00 | 20 |
| donors | male | 8 | 7.25 | 2.60 | 4 | 5.00 | 7.0 | 9.25 | 11 |
| population | female | 193 | 7.99 | 3.32 | 4 | 5.00 | 7.0 | 10.00 | 19 |
| donors | female | 15 | 6.93 | 2.96 | 4 | 5.00 | 6.0 | 8.50 | 14 |
| 45-54 | | | | | | | | | |
| population | male | 218 | 7.74 | 3.32 | 4 | 5.00 | 7.0 | 9.00 | 20 |
| donors | male | 35 | 7.34 | 3.09 | 4 | 5.00 | 6.0 | 9.00 | 16 |
| population | female | 246 | 7.72 | 3.42 | 4 | 5.00 | 7.0 | 10.00 | 18 |
| donors | female | 76 | 7.17 | 3.36 | 4 | 4.00 | 6.0 | 9.00 | 18 |
| 55-64 | | | | | | | | | |
| population | male | 237 | 8.46 | 3.49 | 4 | 6.00 | 8.0 | 11.00 | 20 |
| donors | male | 47 | 6.83 | 2.38 | 4 | 5.00 | 7.0 | 8.00 | 14 |
| population | female | 242 | 8.64 | 3.48 | 4 | 6.00 | 8.0 | 11.00 | 18 |
| donors | female | 72 | 7.82 | 3.90 | 4 | 5.00 | 7.0 | 9.00 | 20 |
| 65-74 | | | | | | | | | |
| population | male | 179 | 8.85 | 3.11 | 4 | 7.00 | 8.0 | 11.00 | 18 |
| donors | male | 29 | 7.79 | 3.85 | 4 | 5.00 | 6.0 | 9.00 | 20 |
| population | female | 160 | 9.15 | 3.32 | 4 | 6.75 | 9.0 | 11.00 | 18 |
| donors | female | 46 | 6.74 | 3.12 | 4 | 4.00 | 5.5 | 9.00 | 16 |
| >74 | | | | | | | | | |
| population | male | 108 | 10.66 | 3.20 | 4 | 8.00 | 10.5 | 13.00 | 20 |
| donors | male | 7 | 8.14 | 4.34 | 4 | 4.50 | 8.0 | 11.00 | 14 |
| population | female | 112 | 11.04 | 3.77 | 4 | 8.00 | 11.0 | 14.00 | 20 |
| donors | female | 14 | 7.57 | 2.85 | 4 | 6.00 | 7.0 | 8.75 | 15 |

M = mean, SD = standard deviation, q25 = lower quartile, q75 = upper quartile

**Mental Fatigue**

| Group | Sex | n | M | SD | Min | q25 | Median | q75 | Max |
| --- | --- | --- | --- | --- | --- | --- | --- | --- | --- |
| 35-44 | | | | | | | | | |
| population | male | 197 | 7.76 | 3.53 | 4 | 4.00 | 7.0 | 10.00 | 20 |
| donors | male | 8 | 9.00 | 4.28 | 4 | 5.00 | 8.5 | 12.50 | 15 |
| population | female | 193 | 8.17 | 3.48 | 4 | 5.00 | 8.0 | 11.00 | 19 |
| donors | female | 15 | 9.00 | 4.63 | 4 | 5.00 | 7.0 | 13.50 | 18 |
| 45-54 | | | | | | | | | |
| population | male | 218 | 7.59 | 3.42 | 4 | 5.00 | 7.0 | 9.00 | 20 |
| donors | male | 35 | 7.37 | 3.34 | 4 | 4.00 | 7.0 | 9.00 | 16 |
| population | female | 246 | 7.75 | 3.52 | 4 | 5.00 | 7.0 | 10.00 | 19 |
| donors | female | 77 | 8.17 | 4.05 | 4 | 5.00 | 7.0 | 10.00 | 20 |
| 55-64 | | | | | | | | | |
| population | male | 237 | 8.03 | 3.58 | 4 | 5.00 | 7.0 | 10.00 | 20 |
| donors | male | 48 | 7.75 | 3.97 | 4 | 4.75 | 6.0 | 10.25 | 19 |
| population | female | 242 | 8.52 | 3.21 | 4 | 6.00 | 8.0 | 11.00 | 20 |
| donors | female | 72 | 8.43 | 4.59 | 4 | 4.00 | 7.5 | 11.25 | 20 |
| 65-74 | | | | | | | | | |
| population | male | 180 | 8.21 | 3.29 | 4 | 6.00 | 8.0 | 10.25 | 18 |
| donors | male | 28 | 7.21 | 4.25 | 4 | 4.00 | 5.0 | 9.25 | 20 |
| population | female | 160 | 8.86 | 3.16 | 4 | 7.00 | 9.0 | 11.00 | 18 |
| donors | female | 46 | 6.46 | 2.71 | 4 | 4.00 | 5.0 | 9.00 | 14 |
| >74 | | | | | | | | | |
| population | male | 108 | 9.62 | 3.51 | 4 | 7.00 | 10.0 | 12.00 | 20 |
| donors | male | 7 | 7.57 | 3.21 | 4 | 5.50 | 7.0 | 9.50 | 12 |
| population | female | 112 | 10.46 | 3.74 | 4 | 8.00 | 11.0 | 13.00 | 20 |
| donors | female | 14 | 7.50 | 4.33 | 4 | 4.25 | 5.0 | 9.75 | 18 |

M = mean, SD = standard deviation, q25 = lower quartile, q75 = upper quartile

**MFI- 20 Sum score**

| Group | Sex | n | M | SD | Min | q25 | Median | q75 | Max |
| --- | --- | --- | --- | --- | --- | --- | --- | --- | --- |
| 35-44 | | | | | | | | | |
| population | male | 197 | 37.87 | 17.37 | 20 | 25.00 | 33.0 | 44.00 | 100 |
| donors | male | 7 | 39.86 | 14.30 | 24 | 29.50 | 34.0 | 51.00 | 60 |
| population | female | 193 | 40.95 | 16.62 | 20 | 27.00 | 37.0 | 52.00 | 87 |
| donors | female | 15 | 41.93 | 16.24 | 21 | 31.00 | 39.0 | 48.00 | 73 |
| 45-54 | | | | | | | | | |
| population | male | 218 | 38.61 | 16.58 | 20 | 26.00 | 34.0 | 46.00 | 100 |
| donors | male | 34 | 38.12 | 15.19 | 20 | 28.00 | 35.0 | 46.00 | 76 |
| population | female | 245 | 39.35 | 16.42 | 20 | 27.00 | 35.0 | 48.00 | 96 |
| donors | female | 71 | 41.85 | 17.09 | 20 | 29.00 | 39.0 | 51.00 | 97 |
| 55-64 | | | | | | | | | |
| population | male | 236 | 42.31 | 17.58 | 20 | 29.00 | 37.0 | 52.25 | 99 |
| donors | male | 46 | 39.35 | 14.28 | 20 | 29.00 | 35.0 | 46.75 | 73 |
| population | female | 242 | 45.31 | 17.03 | 20 | 31.00 | 42.0 | 58.00 | 90 |
| donors | female | 69 | 42.81 | 18.94 | 20 | 27.00 | 39.0 | 55.00 | 88 |
| 65-74 | | | | | | | | | |
| population | male | 180 | 44.99 | 15.63 | 20 | 32.75 | 42.5 | 57.25 | 78 |
| donors | male | 27 | 37.11 | 15.35 | 20 | 24.50 | 32.0 | 47.00 | 69 |
| population | female | 160 | 48.27 | 17.09 | 20 | 34.00 | 47.0 | 61.25 | 93 |
| donors | female | 37 | 35.92 | 14.64 | 20 | 21.00 | 33.0 | 46.00 | 69 |
| >74 | | | | | | | | | |
| population | male | 108 | 56.97 | 15.84 | 20 | 46.00 | 57.0 | 68.00 | 100 |
| donors | male | 7 | 41.86 | 17.18 | 20 | 31.50 | 38.0 | 52.50 | 67 |
| population | female | 112 | 58.93 | 17.77 | 20 | 43.00 | 59.0 | 74.00 | 100 |
| donors | female | 13 | 36.85 | 14.33 | 20 | 27.00 | 33.0 | 44.00 | 65 |

M = mean, SD = standard deviation, q25 = lower quartile, q75 = upper quartile

**Table S7:** Statistics and effect sizes for the Wilcoxon-Mann-Whitney U test for the comparisons of the MFI-20 subscale scores and sum scores between the LKD and the general population sample by age and sex

| Age groups | Male | Female |
| --- | --- | --- |
| **General Fatigue** | | |
| 35-44 | W = 565, p = 0.167, d = -0.603, 95%-CI [-1.31, 0.11] | W = 1038, p = 0.067, d = -0.571, 95%-CI [-1.1, -0.04] |
| 45-54 | W = 3309.5, p = 0.206, d = -0.299, 95%-CI [-0.66, 0.06] | W = 6891, **p < .001***, d = -0.445, 95%-CI [-0.7, -0.19] |
| 55-64 | W = 5375.5, p = 0.705, d = -0.002, 95%-CI [-0.31, 0.31] | W = 9048.5, p = 0.494, d = 0.036, 95%-CI [-0.23, 0.3] |
| 65-74 | W = 3062.5, p = 0.066, d = 0.344, 95%-CI [-0.06, 0.74] | W = 4723.5, **p < .001***, d = 0.671, 95%-CI [0.33, 1.01] |
| >74 | W = 523, p = 0.089, d = 0.794, 95%-CI [0.02, 1.56] | W = 1208.5, **p < .001***, d = 1.267, 95%-CI [0.67, 1.86] |
| **Physical Fatigue** | | |
| 35-44 | W = 607.5, p = 0.589, d = -0.081, 95%-CI [-0.83, 0.67] | W = 1533.5, p = 0.701, d = 0.046, 95%-CI [-0.48, 0.57] |
| 45-54 | W = 3323, p = 0.231, d = -0.22, 95%-CI [-0.58, 0.14] | W = 8151, p = 0.089, d = -0.189, 95%-CI [-0.45, 0.07] |
| 55-64 | W = 5672.5, p = 0.977, d = 0.021, 95%-CI [-0.29, 0.33] | W = 9737.5, p = 0.086, d = 0.191, 95%-CI [-0.07, 0.46] |
| 65-74 | W = 3171, **p = 0.028***, d = 0.453, 95%-CI [0.05, 0.85] | W = 4969, **p < .001***, d = 0.654, 95%-CI [0.32, 0.99] |
| >74 | W = 626.5, **p = 0.004***, d = 1.25, 95%-CI [0.47, 2.03] | W = 1364, **p < .001***, d = 1.431, 95%-CI [0.85, 2.01] |
| **Reduced Activity** | | |
| 35-44 | W = 579.5, p = 0.207, d = -0.106, 95%-CI [-0.81, 0.6] | W = 1678, p = 0.302, d = 0.25, 95%-CI [-0.28, 0.78] |
| 45-54 | W = 4251, p = 0.165, d = 0.246, 95%-CI [-0.12, 0.61] | W = 8872.5, p = 0.428, d = -0.172, 95%-CI [-0.43, 0.08] |
| 55-64 | W = 5918.5, p = 0.465, d = 0.205, 95%-CI [-0.11, 0.52] | W = 9674.5, p = 0.118, d = 0.156, 95%-CI [-0.11, 0.42] |
| 65-74 | W = 3313, **p = 0.02***, d = 0.402, 95%-CI [0.01, 0.8] | W = 4867, **p < .001***, d = 0.751, 95%-CI [0.4, 1.1] |
| >74 | W = 527, p = 0.081, d = 0.719, 95%-CI [-0.05, 1.49] | W = 1272.5, **p < .001***, d = 1.129, 95%-CI [0.55, 1.7] |
| **MFI-Motivation** | | |
| 35-44 | W = 783, p = 0.978, d = 0.089, 95%-CI [-0.62, 0.8] | W = 1723.5, p = 0.217, d = 0.32, 95%-CI [-0.21, 0.85] |
| 45-54 | W = 4083, p = 0.503, d = 0.12, 95%-CI [-0.24, 0.48] | W = 10394.5, p = 0.137, d = 0.161, 95%-CI [-0.1, 0.42] |
| 55-64 | W = 7027, **p = 0.004***, d = 0.489, 95%-CI [0.17, 0.8] | W = 10278.5, **p = 0.02***, d = 0.231, 95%-CI [-0.03, 0.49] |
| 65-74 | W = 3211.5, **p = 0.04***, d = 0.329, 95%-CI [-0.06, 0.72] | W = 5270, **p < .001***, d = 0.736, 95%-CI [0.4, 1.07] |
| >74 | W = 519.5, p = 0.097, d = 0.769, 95%-CI [0, 1.54] | W = 1208.5, **p < .001***, d = 0.943, 95%-CI [0.37, 1.51] |
| **MFI-Mental** | | |
| 35-44 | W = 635, p = 0.349, d = -0.348, 95%-CI [-1.06, 0.36] | W = 1346.5, p = 0.652, d = -0.232, 95%-CI [-0.76, 0.29] |
| 45-54 | W = 3977, p = 0.685, d = 0.065, 95%-CI [-0.29, 0.42] | W = 9078.5, p = 0.58, d = -0.115, 95%-CI [-0.37, 0.14] |
| 55-64 | W = 6168.5, p = 0.353, d = 0.078, 95%-CI [-0.23, 0.39] | W = 9553, p = 0.211, d = 0.024, 95%-CI [-0.24, 0.29] |
| 65-74 | W = 3136, **p = 0.036***, d = 0.289, 95%-CI [-0.11, 0.69] | W = 5257, **p < .001***, d = 0.782, 95%-CI [0.44, 1.12] |
| >74 | W = 500.5, p = 0.152, d = 0.587, 95%-CI [-0.18, 1.35] | W = 1126, **p = 0.008***, d = 0.779, 95%-CI [0.21, 1.34] |
| **MFI-Sum score** | | |
| 35-44 | W = 575, p = 0.457, d = -0.115, 95%-CI [-0.87, 0.64] | W = 1345, p = 0.65, d = -0.059, 95%-CI [-0.58, 0.47] |
| 45-54 | W = 3688.5, p = 0.966, d = 0.03, 95%-CI [-0.33, 0.39] | W = 7797, p = 0.184, d = -0.151, 95%-CI [-0.41, 0.11] |
| 55-64 | W = 5805, p = 0.457, d = 0.174, 95%-CI [-0.14, 0.49] | W = 9331, p = 0.136, d = 0.143, 95%-CI [-0.12, 0.41] |
| 65-74 | W = 3221, **p = 0.006***, d = 0.505, 95%-CI [0.1, 0.91] | W = 4220, **p < .001***, d = 0.742, 95%-CI [0.38, 1.11] |
| >74 | W = 560, **p = 0.034***, d = 0.95, 95%-CI [0.17, 1.72] | W = 1202.5, **p < .001***, d = 1.264, 95%-CI [0.67, 1.86] |

CI = confidence interval

**Table S8:** LKD exceeding the cutoff of one and two SD above the mean score of the general population. Groups that exceed the one SD cutoff also contain donors that exceed the two SD cutoff.

|  |  | **Male** | | | | | **Female** | | | | |
| --- | --- | --- | --- | --- | --- | --- | --- | --- | --- | --- | --- |
| **Age groups** |  | **≥35 - ≤44** | **≥45 - ≤54** | **≥55 - ≤64** | **≥65 - ≤74** | **≥75** | **≥35 - ≤44** | **≥45 - ≤54** | **≥55 - ≤64** | **≥65 - ≤74** | **≥75** |
| LKD N=326  (Missings, N=32) |  | 8  (1) | 35  (1) | 48  (2) | 29  (2) | 8  (1) | 15  (0) | 78  (7) | 74  (5) | 49  (12) | 14  (1) |
| **General Fatigue** | **1 SD** | **11.48** | **11.53** | **12.58** | **12.6** | **14.96** | **12.79** | **12.25** | **13.62** | **14.01** | **15.70** |
| LKD n (%) |  | 4 (50) | 9 (25.7) | 6 (12.5) | 5 (17.2) | 0 (0) | 6 (40) | 18 (23.1) | 16 (21.6) | 0 (0) | 0 (0) |
|  | **2 SD** | **15.25** | **15.11** | **16.45** | **16.08** | **18.42** | **16.75** | **16.07** | **17.54** | **18.00** | **19.51** |
| LKD n (%) |  | 0 (0) | 4 (11.4) | 1 (2.1) | 0 (0) | 0 (0) | 3 (20) | 5 (6.4) | 5 (6.8) | 0 (0) | 0 (0) |
| **Physical Fatigue** | **1 SD** | **11.4** | **11.45** | **12.84** | **13.38** | **17.14** | **11.8** | **11.62** | **13.72** | **14.79** | **17.62** |
| LKD n (%) |  | 1 (12.5) | 8 (22.9) | 10 (20.8) | 1 (3.5) | 0 (0) | 3 (20) | 16 (20.5) | 9 (12.2) | 2 (4.1) | 0 (0) |
|  | **2 SD** | **15.41** | **15.31** | **17.10** | **17.25** | **21.22** | **15.63** | **15.41** | **17.92** | **19.14** | **21.98** |
| LKD n (%) |  | 0 (0) | 3 (8.6) | 0 (0) | 0 (0) | 0 (0) | 1 (6.7) | 4 (5.1) | 3 (4.1) | 0 (0) | 0 (0) |
| **Reduced Activity** | **1 SD** | **11.31** | **11.47** | **12.51** | **13.05** | **15.92** | **11.62** | **11.13** | **12.97** | **13.86** | **16.64** |
| LKD n (%) |  | 0 (0)) | 5 (14.3) | 3 (6.3) | 2 (6.9) | 0 (0) | 2 (13.3) | 16 (20.5) | 11 (14.9) | 3 (6.1) | 0 (0) |
|  | **2 SD** | **15.14** | **15.19** | **16.56** | **16.76** | **19.69** | **15.26** | **14.70** | **17.03** | **17.90** | **21.01** |
| LKD n (%) |  | 0 (0) | 0 (0) | 0 (0) | 1 (3.5) | 0 (0) | 0 (0) | 5 (6.4) | 5 (6.8) | 1 (2.0) | 0 (0) |
| **Red. Motivation** | **1 SD** | **10.97** | **11.06** | **11.95** | **11.97** | **13.86** | **11.31** | **11.14** | **12.12** | **12.47** | **14.81** |
| LKD n (%) |  | 1 (12.5) | 4 (11.4) | 2 (4.2) | 5 (17.2) | 2 (25.0) | 2 (13.3) | 10 (12.8) | 10 (13.5) | 3 (6.1) | 1 (7.1) |
|  | 2 SD | **14.38** | **14.38** | **15.45** | **15.08** | **17.06** | **14.63** | **14.55** | **15.60** | **15.79** | **18.58** |
| LKD n (%) |  | 0 (0) | 1 (2.9) | 0 (0) | 1 (3.5) | 0 (0) | 0 (0) | 2 (2.6) | 5 (6.8) | 1 (2.0) | 0 (0) |
| **Mental Fatigue** | **1 SD** | **11.29** | **11.01** | **11.61** | **11.5** | **13.13** | **11.65** | **11.27** | **11.73** | **12.02** | **14.2** |
| LKD n (%) |  | 3 (37.5) | 4 (11.4) | 10 (20.8) | 5 (17.2) | 0 (0) | 5 (33.3) | 16 (20.5) | 18 (24.3) | 1 (2.0) | 1 (7.1) |
|  | **2 SD** | **14.83** | **14.44** | **15.19** | **14.79** | **16.63** | **15.13** | **14.80** | **14.95** | **15.18** | **17.94** |
| LKD n (%) |  | 1 (12.5) | 1 (2.9) | 3 (6.3) | 1 (3.5) | 0 (0) | 1 (6.7) | 7 (9.0) | 10 (13.5) | 0 (0) | 1 (7.1) |
| **Sumscore** | **1 SD** | **55.24** | **55.18** | **59.89** | **60.62** | **72.81** | **57.57** | **55.77** | **62.35** | **65.36** | **76.70** |
| LKD n (%) |  | 2 (25.0) | 7 (20.0) | 6 (12.5) | 3 (10.3) | 0 (0) | 3 (20.0) | 14 (18.0) | 12 (16.2) | 1 (2.0) | 0(0) |
|  | **2 SD** | **72.61** | **71.76** | **77.47** | **76.24** | **88.65** | **74.19** | **72.18** | **79.38** | **82.45** | **94.47** |
| LKD n (%) |  | 0 (0) | 1 (2.9) | 0 (0) | 0 (0) | 0 (0) | 0 (0) | 6 (7.7) | 4 (5.4) | 0 (0) | 0 (0) |
| LKD N = total number of living kidney donors; LKD n = absolute number of living kidney donors exceeding the respective cut-off within each MFI-20 subscale; (%) = percentage of subjects who exceeded the respective cut-off calculated within each MFI-20 subscale; Missings = missing scores within the age category. SD = Standard Deviation Value. Red. = Reduced | | | | | | | | | | | |

**Table S9:** Interaction effects between age and years since donation on fatigue scores in living kidney donors.

| **MFI - Subscales** | **Estimate** | **std. error** | **p-value** |
| --- | --- | --- | --- |
| General Fatigue | -0.00213 | 0.004405 | 0.599 |
| Physical Fatigue | -0.001461 | 0.003762 | 0.698 |
| Reduced Activity | -0.002466 | 0.003678 | 0.5030 |
| Reduced Motivation | -0.001288 | 0.003210 | 0.68843 |
| Mental Fatigue | -0.001288 | 0.003210 | 0.68843 |
| MFI Sum Score | -0.01015 | 0.01628 | 0.533 |
| This table presents the results of the regression models testing the interaction between age and years since donation for each MFI-20 subscale and the MFI-20 sum score. None of the interaction effects were statistically significant (all p > 0.05). Estimates, standard errors, and p-values are shown for each model. | | | |
